# Patterns and correlates of use of evidence-based interventions to control diabetes by local health departments across the USA

**DOI:** 10.1136/bmjdrc-2018-000558

**Published:** 2018-09-05

**Authors:** Rachel G Tabak, Renee G Parks, Peg Allen, Rebekah R Jacob, Stephanie Mazzucca, Katherine A Stamatakis, Allison R Poehler, Marshall H Chin, Maureen Dobbins, Debra Dekker, Ross C Brownson

**Affiliations:** 1 Prevention Research Center in St Louis, Brown School, Washington University in St Louis, St Louis, Missouri, USA; 2 Department of Epidemiology, College for Public Health and Social Justice, Saint Louis University, St Louis, Missouri, USA; 3 Department of Medicine and Chicago Center for Diabetes Translation Research, University of Chicago, Chicago, Illinois, USA; 4 National Collaborating Centre for Methods and Tools and Health Evidence, McMaster University, Hamilton, Ontario, Canada; 5 National Association of County and City Health Officials (NACCHO), District of Columbia, Washington, USA; 6 Department of Surgery (Division of Public Health Sciences) and Alvin J Siteman Cancer Center, Washington University School of Medicine, Washington University, St Louis, Missouri, USA

**Keywords:** public health, prevention, chronic disease prevention and treatment

## Abstract

**Objective:**

The nearly 3000 local health departments (LHDs) nationwide are the front line of public health and are positioned to implement evidence-based interventions (EBIs) for diabetes control. Yet little is currently known about use of diabetes-related EBIs among LHDs. This study used a national online survey to determine the patterns and correlates of the Centers for Disease Control and Prevention Community Guide-recommended EBIs for diabetes control in LHDs.

**Research design and methods:**

A cross-sectional study was conducted to survey a stratified random sample of LHDs regarding department characteristics, respondent characteristics, evidence-based decision making within the LHD, and delivery of EBIs (directly or in collaboration) within five categories (diabetes-related, nutrition, physical activity, obesity, and tobacco). Associations between delivering EBIs and respondent and LHD characteristics and evidence-based decision making were explored using logistic regression models.

**Results:**

Among 240 LHDs there was considerable variation among the EBIs delivered. Diabetes prevalence in the state was positively associated with offering the Diabetes Prevention Program (OR=1.28 (95% CI 1.02 to 1.62)), diabetes self-management education (OR=1.32 (95% CI 1.04 to 1.67)), and identifying patients and determining treatment (OR=1.27 (95% CI 1.05 to 1.54)). Although all organizational supports for evidence-based decision making factors were related in a positive direction, the only significant association was between evaluation capacity and identifying patients with diabetes and determining effective treatment (OR=1.54 (95% CI 1.08 to 2.19)).

**Conclusion:**

Supporting evidence-based decision making and increasing the implementation of these EBIs by more LHDs can help control diabetes nationwide.

**Significance of this study**

**What is already known about this subject?**

- The nearly 3000 local health departments (LHDs) nationwide are thefrontline of public health and are positioned to implement evidence-based interventions (EBIs) for diabetes control.
- Little is currently known about use of diabetes-related EBIs among LHDs.

**What are the new findings?**

- There is wide variation in evidence-based interventions (EBIs) offered at local health departments(LHDs): half of EBIs offered by ≥80% of the sample, and a quarteroffered by fewer than 60%.
- There are several respondent and LHD characteristics associated with offeringeach of the four diabetes-related EBIs.

**How might these results change the focus of research or clinical practice?**

- Supporting evidence-based decision making, and increasing the implementation of EBIs by more LHDs can help control diabetes nationwide.

## Introduction

Diabetes causes significant morbidity and mortality.^1^ Evidence-based interventions (EBIs) are available to help modify lifestyle behaviors related to diabetes (eg, nutrition and physical activity) and enhance its treatment and management.^2–4^ Tools such as the Community Guide (https://www.thecommunityguide.org/topic/diabetes), What Works for Health, and Cochrane reviews are available to support the use of EBIs to prevent and control diabetes.^5–11^ There is a strong case for the engagement of organizations such as local health departments (LHDs) in diabetes prevention and control.^3 4 12^

The nearly 3000 US LHDs are the ‘frontline’ of public health, and are therefore well positioned to implement EBIs for diabetes control directly and/or in collaboration with other organizations serving the same community.^12 13^ These departments typically receive funding from state and local governments, and engage in surveillance and prevention activities (eg, tuberculosis screening, child and adult immunization provision), as well as activities related to environmental health (eg, inspecting food service establishments and day care centers).^14^ As the threats to public health have changed over time, so have the roles of LHDs.^12 15 16^ Public health departments can and should play an important role in diabetes prevention and management.^3 15 17^ One study found that for each 10% increase in public health spending, diabetes mortality fell by 1.4%.^17^ These gains appear to be due, in part, to collaborations and partnerships within communities to provide needed services and achieve common population health goals.^18 19^ Health departments can extend the reach of healthcare providers and the traditional healthcare system, and are able to provide services to community members who may not otherwise have access to preventive care and health screening due to lack of medical insurance or a feeling of alienation from the medical system.^17^

The National Association of County and City Health Officials (NACCHO) conducts an ongoing survey of LHDs, the National Profile of Local Health Departments, to identify the population-based primary prevention activities available in the communities served by LHDs. In 2016, 34% of LHDs reported screening for diabetes, and 74%, 60%, and 57% indicated they offer population-based primary prevention related to nutrition, physical activity, and chronic disease, respectively.^14^ However, these activities were defined broadly and did not ask about specific EBIs. Despite the critical role LHDs play^18 19^ and the widespread initiatives LHDs provide, limited information is available about the programs offered and whether these are EBIs. Detail is also lacking with regard to how LHDs are delivering these EBIs (ie, directly by the department and/or in collaboration) at the local level. Further, given the documented gap in translation of research to public health practice,^20^ a fuller understanding of factors that facilitate and/or hinder EBI implementation is needed.

Previous research has suggested that organizational processes can impact uptake of EBIs and that the components of evidence-based decision making (EBDM) can support implementation of EBIs.^21–23^ EBDM operates at multiple levels within an LHD and includes summarizing the findings from the best available peer-reviewed evidence, using data and information systems, applying program planning frameworks, engaging the community in assessment and decision making, conducting sound evaluation, and synthesizing science and communication skills with common sense and political acumen for dissemination to other stakeholders and decision makers.^24^ In public health agency settings, management support for EBDM is associated with improved performance.^25^

This study seeks to assess LHDs’ delivery of EBIs related to diabetes prevention and control in several categories (diabetes-related such as the Diabetes Prevention Program (DPP) or self-management education, obesity, physical activity, nutrition and tobacco), and whether these are delivered directly, in collaboration, and/or both. Further, for diabetes-related EBIs, factors at the level of the LHD, including EBDM, associated with delivering each EBI were explored.

## Research design and methods

This cross-sectional survey was part of a larger dissemination study focusing on efforts to improve evidence-based diabetes management and chronic disease control among LHDs.^26^

### Participants and recruitment

LHDs were drawn from the 1677 LHDs across the USA which reported in the 2016 NACCHO National Profile that their agency screens for diabetes or body mass index (BMI), or conducts population-based primary prevention activities for nutrition or physical activity (ie, the National Profile survey asks whether the LHDs ‘screen for diabetes or BMI’ and ‘conduct population-based primary prevention activities for nutrition or physical activity’ directly or via contract). A stratified random sample of 600 LHDs were selected according to three jurisdiction population size categories (small <50 000, medium 50 000–199 999, and large ≥200 000). Efforts were made to distribute the sample across LHD jurisdiction population sizes. Within each selected LHD, the lead practitioner working in chronic disease control was invited to participate in the current study (eg, one participant per health department). After excluding non-valid email addresses, the final recruitment sample was 579.

### Data collection

Survey invitation emails included study information and a link to complete the survey online via the Qualtrics software. To enhance participation, 1 week prior to the survey invitation, a preinvitation email informing survey contacts about the purpose of the study was sent. If a potential participant did not respond to the invitation, follow-up included three reminder emails and two follow-up calls. As compensation for their time completing the survey, respondents were offered a $20 Amazon.com gift card.

### Survey development

Details of the survey development process have been described elsewhere.^26^ Briefly, the survey drew on previous research conducted by the project team^26^ and existing instruments identified through snowball sampling of other researchers’ measures identified by the study team. In addition to three rounds of input, cognitive response testing interviews with 10 practitioners like those in the target audience and an assessment of test–retest reliability were conducted.

### Respondent and LHD characteristics

**Figure 1:**
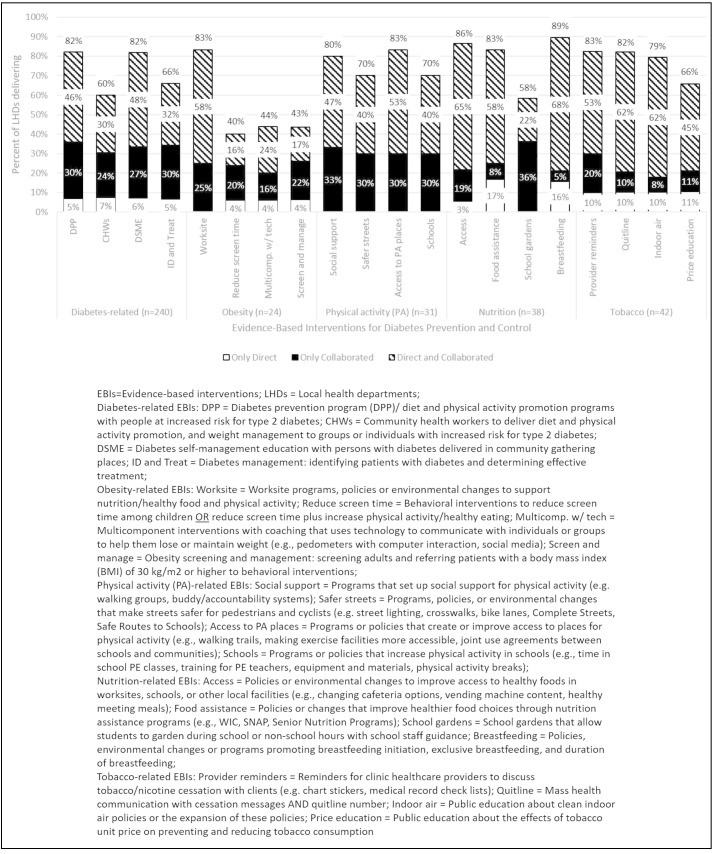
Percentage of LHDs that reported delivering EBIs directly and/or collaboratively with a partnering organization. SNAP, Supplemental Nutrition Assistance Program; WIC program, Women, Infants, and Children program.

Respondents reported the characteristics of their LHD (eg, current status in Public Health Accreditation Board accreditation efforts) and themselves (eg, age group, years in current position); these characteristics are listed in table 1. The survey also included the Short Grit Scale, which measures respondent characteristics: passion and perseverance for long-term goals.^27^ Perceived organizational support for EBDM was assessed using six factors derived from the survey using confirmatory factor analysis (full item wording is available in online supplementary table 1; factor development and validation are described elsewhere^28^). The organizational support for EBDM factors, as shown in Parks *et al* 26 (figure 1), includes awareness of EBDM (three items), capacity for EBDM (seven items), resource availability (three items), evaluation capacity (three items), EBDM climate cultivation (three items), and partnerships to support EBDM (three items).

10.1136/bmjdrc-2018-000558.supp1 Supplementary data

**Table 1:** LHD and respondent characteristics of LHDs in the total sample (n=376)

	n (%*) or mean (SD)
Respondent characteristics	
Age group (years), n (%)	
20–29	14 (3.7)
30–39	86 (23)
40–49	111 (30)
50–59	107 (28)
60+	57 (15)
Race/Ethnicity, n (%)	
White	315 (84.0)
Black/African–American	26 (6.9)
Other race	27 (7.2)
Hispanic or Latino	7 (1.9)
Sex, n (%)	
Male	60 (16)
Female	312 (83)
Master’s degree or higher in any field, n (%)	
No	155 (42)
Yes	216 (58)
Public health master’s or doctorate, n (%)	
No	253 (68)
Yes	118 (32)
Position, n (%)	
Top executive, health director/officer/commissioner	97 (26)
Administrator, deputy or assistant director	77 (20)
Manager of a division or program	138 (37)
Program coordinator	33 (8.8)
Technical expert position (evaluator, epidemiologist, health educator)/other	30 (8.0)
Years in current position (years), n (%)	
<5	202 (54)
5–9	87 (23)
10–19	60 (16)
20+	25 (6.7)
Years in public health (years), n (%)	
<5	41 (11)
5–9	66 (18)
10–19	118 (32)
20+	149 (40)
Short Grit Scale, mean (SD)	4.0 (0.48)
LHD characteristics	
LHD jurisdiction population category, n (%)	
Small (<50 000)	118 (32)
Medium (50 000–199 999)	124 (33)
Large (200 000+)	128 (35)
PHAB-accredited or preparing to apply, n (%)	
Currently accredited	113 (30)
Recently applied but not yet accredited	42 (11)
Yes, but have not yet applied	84 (22)
No	107 (28)
Unsure	29 (7.7)
Currently participate in academic partnerships, n (%)	
Yes	272 (73)
No/Unsure	99 (27)
Diabetes prevalence in the state, mean (SD)	9.2 (1.5)

*% within respondent and LHD characteristic categories.

LHD, local health department; PHAB, Public Health Accreditation Board.

### Assessment of EBIs offered

For the items assessing EBI delivery, sources such as the Community Guide^7^ and What Works for Health^8 9^ were used to identify EBIs, which LHDs might offer either directly or in collaboration. EBIs fell in one of the five categories of diabetes prevention and control activities addressed in the public and community health setting (ie, diabetes-related, obesity, physical activity, nutrition, and tobacco), and were reviewed by the study team to select those with the strongest evidence base. To minimize respondent burden and increase accuracy in reporting, participants were only asked to report on EBIs within a given category (ie, diabetes-related, obesity, physical activity, nutrition, and tobacco), which was determined by the program area in which they reported working (ie, diabetes-related, obesity, physical activity, nutrition, and tobacco). The decision logic was set to increase the sample of participants asked to report on the four diabetes-related EBIs; those who reported diabetes as a program area—whether alone (diabetes only) or in combination with other program areas—were asked to respond to the four diabetes-related EBIs. Thus 240 participants were asked to report on the diabetes-related EBIs, and 24, 31, 38, and 42 participants were asked to report on obesity, physical activity, nutrition, and tobacco EBIs, respectively. Each category included four EBIs and asked participants to report whether their LHD offered the EBI directly, in collaboration with a partner, both (directly/in collaboration), or neither (figure 1 lists the EBIs). The survey defined ‘delivered’ as ‘In the past year, has your agency directly delivered, and has your agency collaborated with organizations to support delivery of the following diabetes interventions’. Collaborated with was defined as ‘served as a community/clinical referral source, or a convener that facilitates the program or referral system’.

### Analysis

Participant and LHD characteristics were summarized using descriptive statistics. Descriptive analyses were also used to describe direct EBI implementation and collaborative implementation (ie, if the EBIs were offered, were they delivered directly by the LHD and/or in collaboration). Given the focus of the study, only the diabetes-related EBIs had a large enough sample size to explore in more depth. Bivariate logistic regression models were used to explore whether LHD characteristics and EBDM scores were associated with whether the LHD offered each diabetes-related EBI and whether the LHD offered all four diabetes-related EBIs. Analyses were performed in SPSS V.24; significance levels for the models were set at p<0.05.

## Results

The 376 responding LHD practitioners (one survey participant per LHD) (65% response rate) were evenly distributed across jurisdiction population size categories; 30% worked for an accredited LHD (table 1). Respondents were primarily female (83%), older than 40 years (73%), and had worked in public health for at least 10 years (72%). In terms of training, 58% of the respondents reported a master’s degree or higher in any field, while 32% reported a master’s or doctorate in public health. Most respondents were a manager of a division or program (37%), the top executive, health director/officer/commissioner (26%), or an administrator, deputy or assistant director (20%) at the LHD. Additional respondent and LHD characteristics are shown in table 1.

There was considerable variation among the diabetes-related EBIs delivered directly and/or in collaboration, with greater than 80% of the respondents reporting they offered the DPP (82%) and diabetes self-management education (81%), compared with 61% offering community health worker programming and 67% offering diabetes screening and treatment referrals (figure 1). Of the 24 LHDs that were asked about obesity EBIs, the only commonly reported EBI was worksite programs, policies or environmental changes to promote nutrition/healthy food and physical activity (83%). Greater than 80% of the 38 LHDs that were asked about the nutrition EBIs reported three of these EBIs were delivered (ie, policies or environmental changes to improve access to healthy foods in worksites, schools, or other local facilities; policies or changes that improve healthier food choices through nutrition assistance programs; and policies, environmental changes or programs promoting breast feeding); school gardens were reported by only 57% of the 38 LHDs. Thirty-one respondents were asked about physical activity promotion EBIs (ie, programs that set up social support for physical activity; programs, policies, or environmental changes that make streets safer for pedestrians and cyclists; programs or policies that create or improve access to places for physical activity; and programs or policies that increase physical activity in schools), and these EBIs were commonly delivered (all ≥70%). Tobacco EBIs were also commonly delivered directly and/or in collaboration, with ≥80% of LHDs delivering each of the three tobacco EBIs and 67% delivering the fourth EBI.

Five EBIs (including all four physical activity EBIs) were only offered in collaboration or both directly/in collaboration with partners, but were not reported to be offered only directly. Most of the remaining EBIs (n=13) were offered only directly by 3%–11% of LHDs asked. Only two EBIs, both nutrition EBIs (improving food choices in assistance programs and promoting breast feeding) were offered only directly at more than 11% of LHDs.

Among the 240 LHDs asked to report on the diabetes-related EBIs, there were several associations between respondent and LHD characteristics, as well as the organizational support for EBDM factors and the EBIs (tables 2 and 3). Most consistently at the respondent level, how long the respondent had been in their current position and their age were both negatively associated with using community health workers to deliver diet and physical activity promotion and/or weight management to groups or individuals with increased risk for type 2 diabetes and with delivering all four diabetes-related EBIs. At the LHD level, diabetes prevalence in the state was associated with offering three of the EBIs: the DPP (OR=1.28 (95% CI 1.02 to 1.62)), diabetes self-management education (OR=1.32 (95% CI 1.04 to 1.67)), and identifying patients and determining treatment (OR=1.27 (95% CI 1.05 to 1.54)). Finally, although all organizational supports for EBDM factors were related in a positive direction with offering the EBIs, the only significant association was between evaluation capacity and identifying patients with diabetes and determining effective treatment (OR=1.54 (95% CI 1.08 to 2.19)).

**Table 2:** LHD and respondent characteristics of LHDs in the sample reporting on diabetes-related EBIs (n=240)

	LHDs offering* diabetes-related EBIs, n (%†) or mean (SE)					
	Total	DPP‡	CHWs§	DSME¶	Identify**	All four
Respondent characteristics						
Age group (years), n (%)						
20–29	9	7 (78)	7 (78)	8 (89)	7 (78)	6 (67)
30–39	57	47 (82)	35 (61)	42 (78)	37 (69)	22 (39)
40–49	73	62 (85)	52 (71)	64 (89)	51 (72)	38 (52)
50–59	67	54 (81)	37 (55)	51 (80)	39 (63)	22 (33)
60+	33	26 (79)	12 (36)	25 (76)	17 (52)	7 (21)
Pearson’s χ^2^ p		0.93	**0.01**	0.36	0.27	**0.01**
Race/Ethnicity, n (%)						
White	203	167 (82)	118 (58)	162 (83)	125 (64)	76 (37)
Black/African–American	17	14 (82)	11 (65)	14 (82)	13 (76)	9 (53)
Other race	14	10 (71)	9 (64)	9 (64)	9 (69)	6 (43)
Hispanic or Latino	5	5 (100)	5 (100)	5 (100)	4 (80)	4 (80)
Pearson’s χ^2^ p		0.54	0.27	0.25	0.67	0.16
Sex, n (%)						
Male	36	31 (86)	22 (61)	31 (91)	25 (78)	16 (44)
Female	202	165 (82)	120 (59)	158 (80)	126 (64)	79 (39)
Pearson’s χ^2^ p		0.52	0.85	0.13	0.12	0.55
Master’s degree or higher in any field (n%)						
No	110	85 (77)	70 (64)	87 (83)	68 (66)	46 (42)
Yes	126	108 (86)	71 (56)	100 (81)	80 (65)	47 (37)
Pearson’s χ^2^ p		0.09	0.25	0.67	0.88	0.48
Public health master’s or doctorate, n (%)						
No	170	139 (82)	105 (62)	138 (84)	109 (67)	70 (41)
Yes	66	54 (82)	36 (55)	49 (77)	39 (61)	23 (35)
Pearson’s χ^2^ p		0.99	0.31	0.21	0.37	0.37
Position, n (%)						
Top executive, health director/officer/commissioner	60	53 (88)	30 (50)	50 (83)	44 (73)	25 (42)
Administrator, deputy or assistant director	53	41 (77)	37 (70)	44 (83)	37 (70)	25 (47)
Manager of a division or program	83	72 (87)	51 (61)	67 (84)	46 (58)	30 (36)
Program coordinator	27	21 (78)	16 (59)	19 (76)	18 (75)	12 (44)
Technical expert position (evaluator, epidemiologist, health educator)/other	16	9 (56)	9 (56)	10 (71)	6 (46)	3 (19)
Pearson’s χ^2^ p		**0.02**	0.31	0.75	0.13	0.29
Years in current position, n (%)						
<5	135	112 (83)	90 (67)	108 (82)	90 (70)	63 (47)
5–9	57	48 (84)	34 (60)	47 (84)	34 (61)	21 (37)
10–19	31	23 (74)	16 (52)	22 (73)	16 (55)	8 (26)
20+	15	13 (87)	3 (20)	13 (87)	11 (73)	3 (20)
Pearson’s χ^2^ p		0.62	**0.00**	0.59	0.34	0.05
Years in public health, n (%)						
<5	28	22 (79)	23 (82)	23 (85)	21 (78)	17 (61)
5–9	38	33 (87)	23 (61)	30 (81)	25 (68)	15 (39)
10–19	81	65 (80)	44 (54)	67 (85)	53 (68)	32 (40)
20+	91	76 (84)	53 (58)	70 (79)	52 (60)	31 (34)
Pearson’s χ2 p		0.77	0.07	0.73	0.34	0.10
Short Grit Scale, mean (SE)						
Not offered		3.96 (0.48)	4.03 (0.49)	4.01 (0.52)	3.95 (0.50)	4.00 (0.48)
Offered		4.00 (0.48)	3.97 (0.46)	3.99 (0.47)	4.01 (0.47)	3.98 (0.48)
Mean difference		−0.04 (0.08)	0.06 (0.06)	0.02 (0.08)	−0.06 (0.07)	0.03 (0.06)
t (p)		−0.48 (0.63)	0.99 (0.32)	0.29 (0.77)	−0.95 (0.34)	0.41 (0.68)
LHD characteristics						
LHD jurisdiction population category, n (%)						
Small (<50 000)	79	58 (73)	45 (57)	62 (81)	48 (63)	30 (38)
Medium (50 000–199 999)	75	64 (85)	42 (56)	60 (82)	51 (71)	32 (43)
Large (200 000+)	84	73 (87)	56 (67)	67 (83)	51 (64)	33 (39)
Pearson’s χ^2^ p		0.05	0.31	0.93	0.55	0.83
PHAB-accredited or preparing to apply, n (%)						
Currently accredited	69	59 (86)	46 (67)	55 (81)	45 (67)	26 (38)
Recently applied but not yet accredited	28	24 (86)	18 (64)	23 (85)	18 (67)	13 (46)
Yes, but have not yet applied	43	36 (84)	26 (60)	34 (85)	21 (55)	16 (37)
No	78	61 (78)	39 (50)	62 (79)	52 (67)	29 (37)
Unsure	21	16 (76)	14 (67)	16 (84)	15 (79)	11 (52)
Pearson’s χ^2^ p		0.71	0.27	0.93	0.49	0.67
Currently participate in academic partnerships, n (%)						
Yes	173	146 (84)	108 (62)	143 (85)	115 (69)	75 (43)
No/Unsure	65	50 (77)	35 (54)	47 (76)	36 (59)	20 (31)
Pearson’s χ^2^ p		0.18	0.23	0.12	0.16	0.08
Diabetes prevalence in the state, mean (SE)						
Not offered		8.91 (1.47)	9.27 (1.52)	8.85 (1.43)	8.98 (1.47)	9.22 (1.43)
Offered*		9.45 (1.51)	9.40 (1.51)	9.45 (1.53)	9.51 (1.51)	9.54 (1.62)
Mean difference		−0.54 (0.25)	−0.13 (0.20)	−0.60 (0.26)	−0.53 (0.21)	−0.31 (0.20)
t (p)		−2.13 (**0.03**)	−0.65 (0.52)	−2.33 (**0.02**)	−2.55 (**0.01**)	−1.58 (0.12)
Organizational support for EBDM (standardized)						
Factor 1: awareness of EBDM, mean (SE)						
Not offered		0.01 (0.13)	0.05 (0.08)	−0.04 (0.12)	−0.06 (0.08)	−0.16 (0.28)
Offered*		0.05 (0.05)	0.05 (0.05)	0.08 (0.05)	0.11 (0.05)	0.06 (0.04)
Mean difference		−0.04 (0.12)	0.00 (0.09)	−0.12 (0.11)	−0.16 (0.09)	−0.22 (0.18)
t (p)		−0.34 (0.73)	0.00 (1.00)	−1.08 (0.28)	−1.77 (0.08)	−1.21 (0.23)
Factor 2: capacity for EBDM, mean (SE)						
Not offered		−0.01 (0.14)	0.03 (0.08)	−0.03 (0.13)	−0.07 (0.09)	−0.21 (0.31)
Offered*		0.06 (0.05)	0.05 (0.06)	0.08 (0.05)	0.11 (0.06)	0.06 (0.05)
Mean difference		−0.06 (0.13)	−0.02 (0.10)	−0.11 (0.12)	−0.18 (0.10)	−0.28 (0.20)
t (p)		−0.51 (0.61)	−0.21 (0.83)	−0.88 (0.38)	−1.80 (0.07)	−1.38 (0.17)
Factor 3: resource availability, mean (SE)						
Not offered		−0.07 (0.11)	0.04 (0.07)	0.00 (0.10)	−0.04 (0.08)	−0.19 (0.24)
Offered*		0.06 (0.05)	0.03 (0.05)	0.06 (0.05)	0.09 (0.05)	0.05 (0.04)
Mean difference		−0.13 (0.11)	0.00 (0.09)	−0.07 (0.11)	−0.14 (0.09)	−0.24 (0.18)
t (p)		−1.13 (0.26)	0.03 (0.98)	−0.59 (0.56)	−1.55 (0.12)	−1.37 (0.17)
Factor 4: evaluation capacity, mean (SE)						
Not offered		−0.08 (0.15)	0.09 (0.09)	−0.12 (0.13)	−0.14 (0.09)	−0.27 (0.33)
Offered*		0.06 (0.06)	0.00 (0.06)	0.08 (0.06)	0.13 (0.06)	0.06 (0.05)
Mean difference		−0.14 (0.14)	0.09 (0.11)	−0.20 (0.14)	−0.27 (0.11)	−0.33 (0.22)
t (p)		−1.02 (0.31)	0.82 (0.42)	−1.47 (0.14)	−**2.47** (**0.01**)	−1.51 (0.13)
Factor 5: EBDM climate cultivation, mean (SE)						
Not offered		0.08 (0.10)	0.08 (0.06)	0.02 (0.09)	−0.04 (0.07)	−0.06 (0.22)
Offered*		0.03 (0.04)	0.01 (0.04)	0.05 (0.04)	0.08 (0.04)	0.04 (0.04)
Mean difference		0.05 (0.09)	0.07 (0.07)	−0.03 (0.09)	−0.13 (0.08)	−0.10 (0.15)
t (p)		0.57 (0.57)	1.01 (0.31)	−0.32 (0.75)	−1.64 (0.10)	−0.69 (0.49)
Factor 6: partnerships to support EBDM, mean (SE)						
Not offered		−0.03 (0.11)	−0.01 (0.07)	−0.01 (0.11)	−0.10 (0.08)	−0.23 (0.25)
Offered*		−0.02 (0.04)	−0.03 (0.05)	−0.01 (0.04)	0.02 (0.05)	−0.01 (0.04)
Mean difference		−0.01 (0.11)	0.01 (0.08)	0.00 (0.11)	−0.12 (0.09)	−0.22 (0.17)
t (p)		−0.13 (0.90)	0.15 (0.88)	0.03 (0.98)	−1.36 (0.17)	−1.31 (0.19)

Bold values indicate statistically significant relationships according to a n alpha=0.05 threshold.

*Each category included four EBIs and asked participants to report whether their LHD offered the EBI directly, in collaboration with a partner, both (directly/in collaboration), or neither.

†% within respondent and LHD characteristic categories.

‡Diet and physical activity promotion programs with people at increased risk for type 2 diabetes, such as the Diabetes Prevention Program (DPP).

§Community health workers (CHWs) to deliver diet and physical activity promotion and weight management to groups or individuals with increased risk for type 2 diabetes.

¶Diabetes self-management education (DSME) with persons with diabetes delivered in community gathering places.

**Diabetes management interventions identifying patients with diabetes and determining effective treatment (identify).

EBDM, evidence-based decision making; EBIs, evidence-based interventions; LHDs, local health departments; PHAB, Public Health Accreditation Board.

**Table 3:** Associations between respondent and LHD characteristics and delivering diabetes-related EBIs directly or in collaboration

	DPP*	CHWs†	DSME‡	Identify§	Offering all 4 diabetes EBIs
	OR (95% CI)	OR (95% CI)	OR (95% CI)	OR (95% CI)	OR (95% CI)
Respondent characteristics					
Master’s degree or higher in any field	1.76 (0.90 to 3.45)	0.74 (0.44 to 1.25)	0.86 (0.44 to 1.69)	0.96 (0.55 to 1.66)	0.83 (0.49 to 1.40)
Public health master’s or doctorate	1.00 (0.48 to 2.10)	0.74 (0.42 to 1.32)	0.64 (0.31 to 1.30)	0.76 (0.42 to 1.38)	0.76 (0.42 to 1.38)
Position (top executive, health director, health officer, commissioner=referent)	**0.74 (0.56 to 0.99)**	1.07 (0.86 to 1.33)	0.87 (0.65 to 1.15)	0.82 (0.64 to 1.04)	0.86 (0.69 to 1.08)
Years in current position	0.93 (0.66 to 1.33)	**0.62 (0.46 to 0.82)**	0.94 (0.66 to 1.34)	0.88 (0.66 to 1.17)	**0.65 (0.47 to 0.88)**
Years in public health	1.04 (0.75 to 1.45)	0.78 (0.59 to 1.01)	0.88 (0.62 to 1.24)	0.78 (0.59 to 1.04)	**0.75 (0.58 to 0.97)**
Age	0.94 (0.69 to 1.28)	**0.71 (0.55 to 0.91)**	0.89 (0.66 to 1.22)	0.78 (0.60 to 1.01)	**0.72 (0.56 to 0.93)**
Race/Ethnicity	0.99 (0.60 to 1.63)	1.44 (0.92 to 2.24)	0.88 (0.55 to 1.40)	1.26 (0.80 to 1.98)	1.44 (0.97 to 2.13)
Sex	0.72 (0.26 to 1.97)	0.93 (0.45 to 1.93)	0.39 (0.11 to 1.35)	0.50 (0.20 to 1.21)	0.80 (0.39 to 1.64)
Short Grit Scale	1.19 (0.59 to 2.38)	0.76 (0.44 to 1.31)	0.90 (0.44 to 1.83)	1.32 (0.75 to 2.33)	0.89 (0.52 to 1.54)
LHD characteristics					
Jurisdiction population categories (<50 000=referent)	**1.59 (1.05 to 2.40)**	1.23 (0.90 to 1.68)	1.08 (0.72 to 1.61)	1.01 (0.73 to 1.41)	1.03 (0.75 to 1.40)
PHAB accreditation status	0.84 (0.66 to 1.08)	0.87 (0.72 to 1.05)	0.99 (0.78 to 1.26)	1.04 (0.85 to 1.26)	1.04 (0.86 to 1.25)
Academic partnership	0.62 (0.30 to 1.25)	0.70 (0.39 to 1.25)	0.57 (0.28 to 1.17)	0.65 (0.36 to 1.19)	0.58 (0.32 to 1.07)
Diabetes prevalence in the state	**1.28 (1.02 to 1.62)**	1.06 (0.89 to 1.26)	**1.32 (1.04 to 1.67)**	**1.27 (1.05 to 1.54)**	1.15 (0.97 to 1.36)
Organizational support for EBDM					
Factor 1: awareness of EBDM	1.09 (0.67 to 1.75)	1.00 (0.69 to 1.45)	1.31 (0.80 to 2.16)	1.45 (0.96 to 2.20)	1.59 (0.75 to 3.37)
Factor 2: capacity for EBDM	1.12 (0.72 to 1.74)	1.04 (0.74 to 1.46)	1.23 (0.78 to 1.95)	1.42 (0.97 to 2.09)	1.62 (0.81 to 3.22)
Factor 3: resource availability	1.33 (0.81 to 2.19)	0.99 (0.67 to 1.47)	1.17 (0.70 to 1.96)	1.40 (0.91 to 2.15)	1.72 (0.79 to 3.76)
Factor 4: evaluation capacity	1.23 (0.83 to 1.83)	0.88 (0.64 to 1.20)	1.36 (0.90 to 2.05)	1.54 (1.08 to 2.19)	1.60 (0.87 to 2.96)
Factor 5: EBDM climate cultivation	0.84 (0.46 to 1.53)	0.79 (0.49 to 1.26)	1.10 (0.60 to 2.02)	1.52 (0.92 to 2.51)	1.38 (0.55 to 3.47)
Factor 6: partnerships to support EBDM	1.03 (0.62 to 1.73)	0.97 (0.65 to 1.45)	0.99 (0.59 to 1.68)	1.34 (0.88 to 2.06)	1.65 (0.78 to 3.48)

OR from unadjusted bivariate model.

Bold values indicate statistically significant relationships according to a n alpha=0.05 threshold.

*Diet and physical activity promotion programs with people at increased risk for type 2 diabetes, such as the Diabetes Prevention Program (DPP).

†Community health workers (CHWs) to deliver diet and physical activity promotion and weight management to groups or individuals with increased risk for type 2 diabetes.

‡Diabetes self-management education (DSME) with persons with diabetes delivered in community gathering places.

§Diabetes management interventions identifying patients with diabetes and determining effective treatment (identify).

EBDM, evidence-based decision making; EBI, evidence-based intervention; LHD, local health department; PHAB, Public Health Accreditation Board.

## Discussion

This study found in a national sample of LHDs a wide variation in EBI offerings by category of EBI (ie, obesity vs physical activity) and by individual EBI, with half of the EBIs offered by at least 80% of the reporting LHDs. Widespread adoption of EBIs in public health practice is an encouraging development for effective prevention and management of diabetes. The results demonstrate that collaboration with other organizations in the community appears to be critical to offering EBIs; very few EBIs were offered only directly by the LHD. Offering healthier food assistance programs and breastfeeding promotion were the EBIs with the greatest percentage only being delivered directly by the LHD (17% and 16%, respectively). These may be thought to be more traditional functions of public health.^12 15 16 29^ However, when branching out to the other types of EBIs, with more environment and policy focus, LHDs reported collaboration to accomplish implementation.

Although half of the EBIs were offered by ≥80% of the sample, a quarter of the EBIs were offered by fewer than 60%. Behavioral interventions to reduce screen time; multicomponent interventions with coaching that uses technology to aid in weight loss or maintenance (eg, pedometers with computer interaction, social media); and school gardens are more newly recommended interventions, which may be, in part, why fewer LHDs reported offering these interventions than more conventional programs such as diabetes self-management education or diet and physical activity promotion programs with people at increased risk for type 2 diabetes, such as the DPP. For example, the oldest reference on the What Works for Health web page for school gardens is from 2005.^30^ Screening for obesity in adults and referring those with elevated BMI (>30 kg/m^2^) to behavioral interventions may be offered in a smaller percentage of responding LHDs, as this type of programs may be viewed as more of a clinical service, particularly as the recommendation from the US Preventive Services Task Force is focused on clinicians in primary care settings.^31^ There may be additional barriers to offering interventions where community health workers deliver diet and physical activity promotion and weight management to those with increased risk for type 2 diabetes, such as licensure, cost/turnover, and fears of deportation.^32 33^

Several factors were found to be related to offering each of the diabetes-related EBIs and all four of the diabetes-related EBIs. At the individual level, older respondents and those who had been in their position longer (likely correlated factors) were less likely to report their LHD offered the EBIs. Previous studies have found that perceptions of public health practice models, such as coordinated chronic disease prevention, vary with duration in a state health department^34^; however, while one study found barriers to EBDM to be ranked higher by older practitioners,^21^ another study found older respondents reported higher levels of organizational support for EBDM.^35^ It is possible that older LHD staff are further removed from training, as has been seen in healthcare,^36–38^ or prefer to rely more heavily on learned experience than evidence-based resources when selecting interventions to implement. At the organizational level, the size of the jurisdiction served was positively associated with delivering diet and physical activity promotion programs with people at increased risk for type 2 diabetes, such as the DPP. A pilot study of LHDs in Missouri found organizational characteristics such as LHD size and accreditation status were positively associated with delivering EBIs.^39^ While this cross-sectional study does not allow for assessment of causation, it is notable that, at the LHD level, there was a positive association between diabetes prevalence in the state and offering several of the EBIs. This suggests that higher diabetes prevalence may elevate the issue of diabetes as a priority, and LHDs and their partners may respond with additional EBIs; alternately, higher diabetes prevalence may lead to more funding from the Centers for Disease Control and Prevention. Zhang *et al* 12 found diabetes prevalence to be associated with LHDs screening for diabetes, but not with delivery of obesity prevention programs.

This study provides support for the positive association between organizational support for EBDM and LHDs delivering EBIs. Although all of the organizational supports for EBDM factors were positively associated with offering the EBIs, the only significant association was between evaluation capacity and identifying patients with diabetes and determining effective treatment. This aligns with previous research, which has shown the importance of organizational-level factors related to EBDM and use of research evidence. For example, a pilot study in Missouri LHDs found delivering EBIs to be associated with the perception that the agency gives incentives and rewards to help employees use EBDM principles.^39^ There is a growing literature that capacity for EBDM can be built with sustained efforts (eg, training, technical assistance).^40^

There are limitations to this study, including the sample size; respondents were only asked about EBIs in one category, so only the four items in the diabetes-related EBI category had items with adequate sample size. Future work could explore EBIs in the other categories (eg, obesity, nutrition) to identify whether these associations were significant and whether LHDs might be offering other interventions, which may not have had as strong of an evidence base at the time the EBIs were selected. While this was a national study with LHDs from 44 states and a balance of LHDs by jurisdiction population size, only LHDs that offered some diabetes-related services were included; thus, the findings cannot be generalized to other public health settings such as state health departments or community-based organizations or to all LHDs. While there are no directly comparable data at the national level, the NACCHO National Profile of Local Health Departments, an ongoing survey of LHDs, asked whether population-based primary prevention activities (defined broadly, rather than asking about specific EBIs as in the current study) were performed by the LHD directly, contracted out by the LHD, provided by others in the community independent of LHD funding, or not available in the community.^41^ The 2016 National Profile found a similar percent of LHDs reported programming nutrition (current sample: 97% offer; NACCHO sample: 97% offer), physical activity (current sample: 99% offer; NACCHO sample: 94% offer), and tobacco (current sample: 98% offer; NACCHO sample: 96% offer) were available in their community as was found in the current sample. This suggests the current sample of LHDs is likely representative of those nationwide. Other important limitations include that data were self-reported and there was only one response per LHD. It is possible that LHDs over-reported offering EBIs due to social desirability bias; however, the range of offerings suggests that respondents were willing to report that their LHD did not offer specific EBIs. The self-report nature of the data collection also makes it difficult to interpret how respondents conceptualized delivering EBIs in collaboration, where there might be less knowledge of specific EBI delivery.

The current study highlights important strengths and gaps in EBI offerings in LHDs and identified correlates at the respondent and LHD levels, as well as correlates related to EBDM that are associated with offering diabetes-related EBIs. While many of the characteristics are non-modifiable (ie, age, jurisdiction population category), it is possible to modify EBDM within an LHD.^42 43^ Future work could conduct dissemination and implementation studies to better tease out causality, and to determine whether improvements in EBDM support and capacity can lead to increased offering of EBIs by LHDs, which is critical to addressing diabetes in the US and other countries.
