# Supplementary material for: Patterns and correlates of use of evidence-based interventions to control diabetes by local health departments across the USA
Source: BMJ Open Diabetes Res Care. 2018 Sep 5;6(1):e000558. doi: 10.1136/bmjdrc-2018-000558 (PMC6135437; doi:10.1136/bmjdrc-2018-000558)
Supplement: Supplementary data [file bmjdrc-2018-000558supp001.docx]

Appendix 1. Additional Table

Supplementary Table A-1. Factors and Items Measuring Organizational Supports for Evidence-Based Decision Making (EBDM)

| **Factor 1: Awareness of EBDM** |
| --- |
| I am provided the time to identify evidence-based programs and practices. |
| My direct supervisor recognizes the value of management practices that facilitate EBDM. |
| My work group/division offers employees opportunities to attend evidence-based-decision making trainings. |
| **Factor 2: Capacity for EBDM** |
| I use EBDM in my work. |
| My direct supervisor expects me to use EBDM. |
| My performance is partially evaluated on how well I use EBDM in my work. |
| My work group/division currently has the resources (e.g. staff, facilities, partners) to support application of EBDM. |
| The staff in my work group/division has the necessary skills to carry out EBDM. |
| The majority of my work group/division’s external partners support use of EBDM. |
| Top leadership in my agency encourages use of EBDM. |
| **Factor 3: Resource Availability** |
| Informational resources (e.g. academic journals, guidelines, and toolkits) are available to my work group/division to promote the use of EBDM. |
| My work group/division engages a diverse external network of partners that share resources to facilitate EBDM. |
| Stable funding is available for EBDM. |
| **Factor 4: Evaluation Capacity** |
| My work group/division plans for evaluation of interventions prior to implementation. |
| My work group/division uses evaluation data to monitor and improve interventions. |
| My work group/division distributes intervention evaluation findings to other organizations that can use our findings. |
| **Factor 5: EBDM Climate Cultivation** |
| Information is widely shared in my work group/division so that everyone who makes decisions has access to all available knowledge. |
| My agency is committed to hiring people with relevant training or experience in public health core disciplines (e.g. epidemiology, health education, environmental health). |
| My agency has a culture that supports the processes necessary for EBDM. |
| **Factor 6: Partnerships to Support EBDM** |
| It is important to my agency to have partners who share resources (money, staff time, space, materials). |
| It is important to my agency to have partners in healthcare to address population health issues. |
| It is important to my agency to have partners in other sectors (outside of health) to address population health issues. |

Response options for all items: Strongly Disagree = 1; Strongly Agree=7
